# Supplementary material for: Genomic architecture and prediction of censored time-to-event phenotypes with a Bayesian genome-wide analysis
Source: Nat Commun. 2021 Apr 20;12:2337. doi: 10.1038/s41467-021-22538-w (PMC8058085; doi:10.1038/s41467-021-22538-w)
Supplement: Supplementary file 1 — Supplementary Information [file 41467_2021_22538_MOESM1_ESM.pdf]

Genomic architecture and prediction of censored time-to-event phenotypes with a Bayesian genome-wide analysis

Ojavee et al.

Supplementary Note

Generating data from Generalised gamma distribution

We generate the time-to-event phenotypes Y_i that follows Generalised gamma distribution using the following formula:

$$\log Y_i = \mu + x_i\beta + cw_i - cEw_i, \tag{1}$$

where w_i (random error) has the following density:

$$f(w) = \frac{|\theta|(\theta^{-2}\exp(\theta w))^{\frac{1}{\theta^2}}\exp(-\theta^{-2}\exp(\theta w))}{\Gamma(\frac{1}{\theta^2})}.$$

This specification means that $E[\log(Y_i|\mu + x_i\beta)] = \mu + x_i\beta$. We will specify the constant c so that the phenotype $\log(Y_i)$ would have a fixed heritability h^2:

$$h^2 = \frac{\sigma_G^2}{\sigma_G^2 + c^2 Var(w_i)} \iff c = \sqrt{\Big(\frac{1}{h^2} - 1\Big)\frac{\sigma_G^2}{Var(w_i)}}. \tag{2}$$

For each value of θ we calculate the values of $E(w_i)$ and $Var(w_i)$. The value $\theta = 0$ is not a valid parameter for the Generalised gamma distribution. Therefore, the notation $\theta = 0$ stands for the limiting distribution in the process $\theta \to 0$. It is known that the limiting distribution is the log-normal distribution. If $\theta = 1$ then Y_i has a Weibull distribution.

Generating data with fixed proportion of censored individuals

Suppose that time of censoring is C_i for individual i. The observed event is defined as $T_i = \min(Y_i, C_i)$, where Y_i is the true time of event which in reality can be unobserved. The time of censoring is simulated from uniform distribution $C_i \sim U(0, \tau)$. The parameter of the uniform distribution τ is chosen such that the proportion of censored individuals p_τ (that is for whom $C_i < Y_i$) would be some fixed constant. The choice of τ will be dependent on the distribution of Y_i.

Such generative model guarantees that the censoring times C_i and the event times Y_i are independent which is one of the assumptions of our survival model.

Therefore, the τ is chosen such that we would get a fixed censoring level p_τ:

$$p_\tau = E_I[P(C_I < Y_I | I = i)],$$

where I denotes a random individual.

Proofs of log-concavity

The functions g under investigation are twice differentiable. Therefore, to prove the concavity of g it is sufficient to show that $g''(x) \leq 0$ for every x in the domain of g.

Log-concavity for the posterior of α. As constants do not affect concavity, it is sufficient to show that the following function is concave where $\alpha > 0$.

$$g(\alpha) = \log p(\alpha|D, \mu, \beta) - const =$$

$$(\alpha_0 + d - 1)\log\alpha + \alpha[\sum_{i=1}^n d_i(\log y_i - \mu - x_i\beta) - \kappa_0] + e^{-K}\sum_{i=1}^n[\exp(\alpha(\log(a_i) - \mu - x_i\beta)) - \exp(\alpha(\log(y_i) - \mu - x_i\beta))] =$$

$$A\log\alpha + B\alpha + e^{-K}\sum_{i=1}^n[\exp(\alpha(\log(a_i) - \mu - x_i\beta)) - \exp(\alpha(\log(y_i) - \mu - x_i\beta))] =$$

$$A\log\alpha + B\alpha + e^{-K}\sum_{i=1}^n[\exp(\alpha C_i) - \exp(\alpha D_i)]$$

We see that $A > 0$ and as failure or censoring happens after left truncation then $D_i > C_i$ for every i. The second derivative of g is

$$g''(\alpha) = -\frac{A}{\alpha^2} + e^{-K} \sum_{i=1}^{n} [C_i^2 \exp(\alpha C_i) - D_i^2 \exp(\alpha D_i)].$$

As $A > 0$ and $D_i > C_i$ for every i, then $g''(\alpha) < 0$ for every $\alpha > 0$.

Log-concavity for the posterior of β_j. We need to show that the following function is concave

$$g(\beta_j) = \log p(\beta_j | D, \alpha, \mu, \beta_{-j}) - const =$$
$$-\alpha\beta_j \sum_{i=1}^{n} d_i x_{ij} + \exp(-K) \sum_{i=1}^{n} [\exp(\alpha(\log(a_i) - \mu - x_i\beta)) - \exp(\alpha(\log(y_i) - \mu - x_i\beta))] - \frac{1}{2\sigma_\beta^2}\beta_j^2 =$$
$$-A\beta_j - B\beta_j^2 + \sum_{i=1}^{n} (C_i - D_i) \exp\{-\alpha x_{ij}\beta_j\}.$$

We see that $B > 0$, and $D_i > C_i$, $i \in \{1, ..., n\}$. The second derivative is

$$g''(\beta_j) = -B + \alpha^2 \sum_{i=1}^{n} (C_i - D_i) x_{ij}^2 \exp(-\alpha x_{ij}\beta_j)$$

and clearly $g''(\beta_j) < 0$ for every $\beta_j \in \mathbb{R}$.

Log-concavity for the posterior of δ_q. Analogous to the case of β_j.

Log-concavity for the posterior of μ.

$$g(\mu) = \log p(\mu | D, \alpha, \beta) - const = -\alpha\mu d +$$
$$\exp(-K) \sum_{i=1}^{n} [\exp(\alpha(\log(a_i) - \mu - x_i\beta)) - \exp(\alpha(\log(y_i) - \mu - x_i\beta))] - \frac{1}{2\sigma_\mu^2}\mu^2 =$$
$$-A\mu - B\mu^2 + \sum_{i=1}^{n} (C_i - D_i) \exp\{-\alpha\mu\}$$

We see that $B > 0$, and $D_i > C_i$, $i \in \{1, ..., n\}$. The second derivative is

$$g''(\mu) = -B + \alpha^2 \sum_{i=1}^{n} (C_i - D_i) \exp(-\alpha\mu)$$

and clearly $g''(\mu) < 0$ for every $\mu \in \mathbb{R}$.

SNP heritability of age-at-onset on the log and original scale

We will derive expressions for SNP heritability on the log-scale and on the original scale given that the phenotype follows a Weibull distribution. The quantity defined here is meaningful in terms of heritability if the appearance of the event is guaranteed for all of the individuals (for example menopause but not diagnosis of T2D). For the events that are not guaranteed to happen we call this quantity pseudo-heritability because then the underlying random variable can be improper.

The Weibull data for the model is generated using following expression

$$\log Y_i = \mu + x_i\beta + \frac{w_i}{\alpha} + \frac{K}{\alpha}$$

where w_i (random error) comes from the standard extreme value distribution (Gumbel distribution) and K is Euler-Mascheroni constant. This guarantees that Y_i has a Weibull distribution, $E(\log(Y_i)|\mu + x_i\beta) = \mu + x_i\beta$ and $Var(\log(Y_i)|\mu + x_i\beta) = \frac{\pi^2}{6\alpha^2}$. In the following, we denote $g_i = x_i\beta$ and we assume $g_i \sim N(0, \sigma_G^2)$ where σ_G^2 is the genetic variance of the logarithmed phenotype.

We require the variance of $\log Y_i$ and the corresponding heritability $h^2_{\log}$. Using the law of total variance it is possible to separate the variance components in the following way where the first part represents the genetic variance and second is the error variance.

$$Var(\log Y_i) = E[Var(\mu + g_i + \frac{w_i}{\alpha} + \frac{K}{\alpha}|w_i)] + Var[E(\mu + g_i + \frac{w_i}{\alpha} + \frac{K}{\alpha}|w_i)] = \sigma_G^2 + Var(\mu + \frac{K}{\alpha} + \frac{w_i}{\alpha}) = \sigma_G^2 + \frac{\pi^2}{6\alpha^2} \quad (3)$$

As the genetic variance is σ_G^2 we get the log-scale heritability by dividing the genetic variance component by the total variance:

$$h^2_{\log} = \frac{\sigma_G^2}{\sigma_G^2 + \frac{\pi^2}{6\alpha^2}}. \quad (4)$$

Using the same idea we decompose the variance of Y_i and find the corresponding heritability h^2_{orig}. In addition we need three following results:

Firstly we see that $[\exp(\mu + \frac{K}{\alpha})]^2$ will cancel out when calculating the heritability

$$Var(Y_i) = Var[\exp(\mu + g_i + \frac{w_i}{\alpha} + \frac{K}{\alpha})] = [\exp(\mu + \frac{K}{\alpha})]^2 Var[\exp(g_i + \frac{w_i}{\alpha})]. \quad (5)$$

Secondly, we note that as g_i is normally distributed then $\exp(g_i)$ has a corresponding log-normal distribution and therefore $Var(\exp(g_i)) = [\exp(\sigma_g^2) - 1]\exp(\sigma_g^2)$ and $E(\exp(g_i)) = \exp(\sigma_G^2/2)$.

Thirdly, we see that if w_i has standard extreme value distribution then for $\alpha > 2$

$$E[\exp(\frac{2w_i}{\alpha})] = \Gamma(1 - \frac{2}{\alpha}),$$

otherwise the expected value is undefined and for $\alpha > 1$

$$E[\exp(\frac{w_i}{\alpha})] = \Gamma(1 - \frac{1}{\alpha}),$$

otherwise the expected value is undefined.

Using the law of total variance the genetic and error variance can be separated as

$$Var[\exp(g_i + \frac{w_i}{\alpha})] = E[Var(\exp(g_i + \frac{w_i}{\alpha})|w_i)] + Var[E(\exp(g_i + \frac{w_i}{\alpha})|w_i)]. \quad (6)$$

The genetic variance component is

$$E[Var(\exp(g_i + \frac{w_i}{\alpha})|w_i)] = E[(\exp(\frac{w_i}{\alpha}))^2 Var(\exp(g_i))] = E[\exp(\frac{2w_i}{\alpha})]Var(\exp(g_i)) = \Gamma(1 - \frac{2}{\alpha})[\exp(\sigma_G^2) - 1]\exp(\sigma_G^2). \quad (7)$$

The error variance component is

$$Var[E(\exp(g_i + \frac{w_i}{\alpha})|w_i)] = Var[\exp(\frac{w_i}{\alpha})E(\exp(g_i))] = \left(\exp\left(\frac{\sigma_g^2}{2}\right)\right)^2 Var\left(\exp\left(\frac{w_i}{\alpha}\right)\right) = \exp(\sigma_G^2)\left(\Gamma(1 - \frac{2}{\alpha}) - \left(\Gamma(1 - \frac{1}{\alpha})\right)^2\right). \quad (8)$$

Therefore, by dividing the genetic variance component with the sum of genetic and error variance, we get that the heritability on the original scale, given $\alpha > 2$, is

$$h^2_{orig} = \frac{\Gamma(1-\frac{2}{\alpha})(\exp(\sigma^2_G)-1)}{\Gamma(1-\frac{2}{\alpha})\exp(\sigma^2_G) - \left(\Gamma(1-\frac{1}{\alpha})\right)^2}. \tag{9}$$

	Time to CAD	Time to HBP	Time to Menarche	Time to Menopause	Time to T2D
**All**					
N_{uncens}/N	17,452/360,715	95,123/371,878	200,493/200,493	108,120/151,472	15,813/372,280
Uncensored %	4.8%	25.6%	100%	71.4%	4.2%
Mean (sd)	54.0 (8.86)	51.1 (9.96)	13.0 (1.60)	50.4 (4.28)	54.4 (9.99)
Median (Range)	55.2 (0.0-78.2)	52.0 (18.0-81.0)	13.0 (5.0-25.0)	51.0 (33.0-68.0)	56.3 (0.5-79.3)
**Female**					
N_{uncens}/N	4746/197,471	43,457/199,499	200,493/200,493	108,120/151,472	5587/201,933
Uncensored %	2.4%	21.8%	100%	71.4%	2.8%
Mean (sd)	54.7 (9.03)	50.5 (10.83)	13.0 (1.60)	50.4 (4.28)	54.8 (9.75)
Median (Range)	56.3 (1.5-78.1)	52.0 (18.0-81.0)	13.0 (5.0-25.0)	51.0 (33.0-68.0)	56.3 (0.5-79.3)
**Male**					
N_{uncens}/N	12,706/163,244	51,666/172,379			10,226/170,347
Uncensored %	7.8%	30.0%			6.0%
Mean (sd)	53.7 (8.78)	51.6 (9.14)			54.2 (10.11)
Median (Range)	55.2 (0.0-78.2)	52.0 (18.0-79.0)			55.6 (0.5-79.3)

Table S1. Descriptive statistics for the five UK Biobank phenotypes used in the analysis. Descriptive statistics listed are the number of uncensored individuals N_{uncens}, the number of individuals N, the percentage of uncensored individuals, the mean and the standard deviation across uncensored individuals, the median and the range across uncensored individuals.

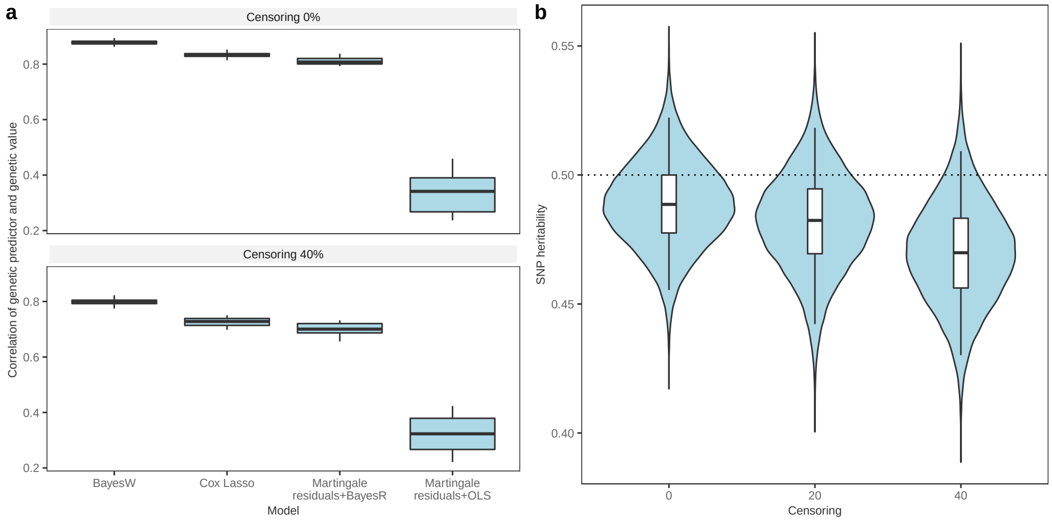

Figure S1. Simulation results for correlated data. 25 simulations used genotypic data from UK biobank randomly chosen individuals and chromosome 22: $N = 20,000$ individuals, $M = 194,922$ markers, $p = 2,000$ causal markers, Weibull phenotype, variance components (0.001,0.01), heritability $h^2 = 0.5$. The independent data set consisted of another 2,000 randomly chosen UK Biobank individuals. (a) Prediction accuracy when predicting to an independent data set across four methods given different censoring levels. Similarly to figure 1 we see that also for the correlated markers BayesW gives us higher accuracy for prediction. The higher level of censoring mildly decreases the prediction accuracy; (b) SNP heritability estimates given the censoring level. Similarly to the uncorrelated case in figure 1 we see that the true heritability falls into the 95% credibility interval. Higher censoring values mildly decrease the power and therefore also the heritability estimate. In panels (a)-(b), bounds of the box show the interquartile range, centre shows the median and minimum and maximum indicate the 95% credibility interval. Source data are provided as a Source Data file.

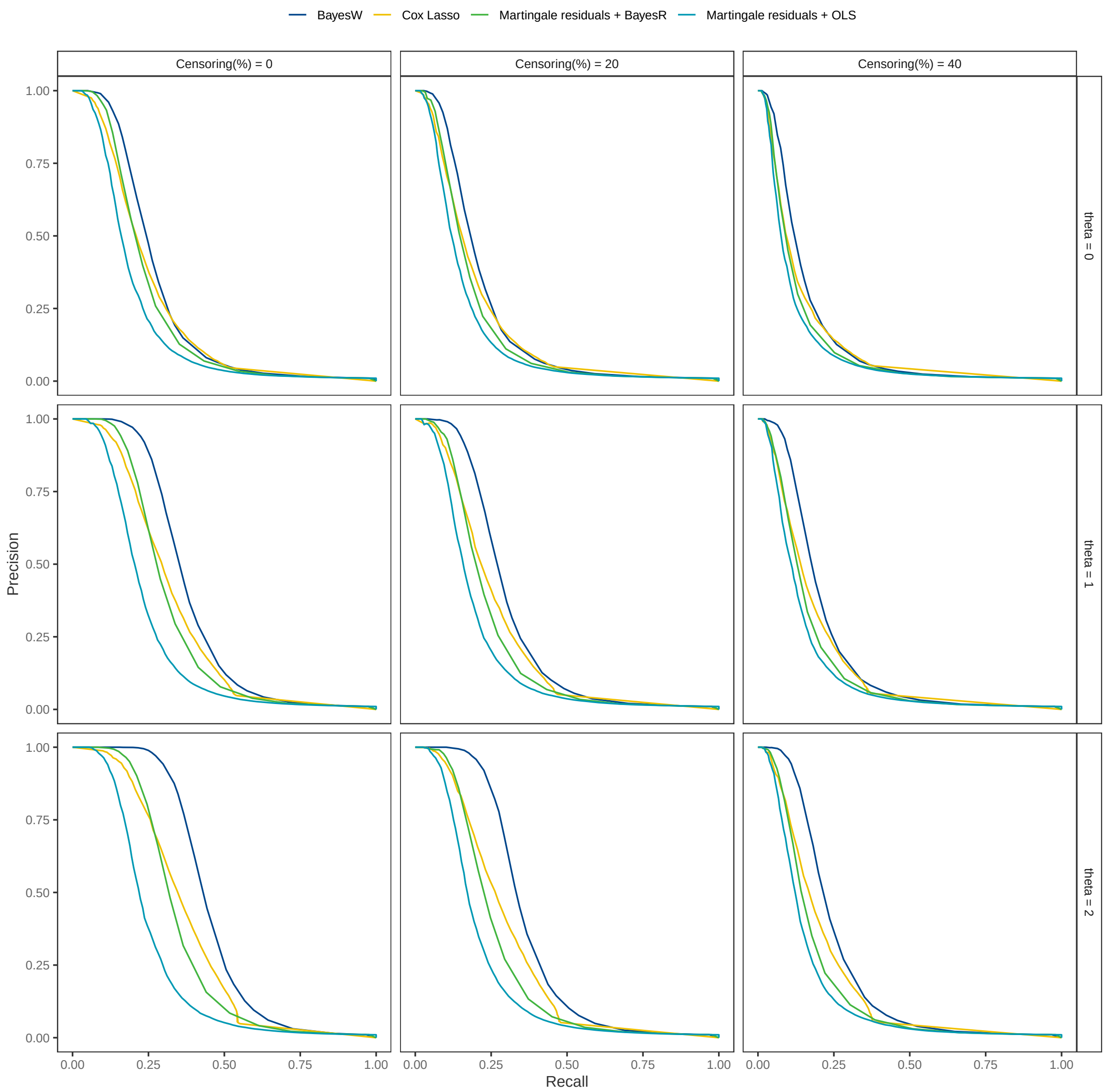

Figure S2. Precision recall curve for different phenotypic distributions and censoring levels. Phenotypes were created by varying the θ parameter of generalised gamma distributions parameter and 25 simulations were run. $\theta = 1$ corresponds to Weibull data and $\theta \to 0+$ (denoted with 0) corresponds to log-normal distribution. Simulation setting: $N = 5,000$ individuals, $M = 50,000$ uncorrelated markers, $p = 500$ causal markers, heritability $h^2 = 0.5$, variance components (0.01). Even for the phenotypes where $\theta \neq 1$, BayesW gets higher precisions for most of the recall values which indicates that the model is relatively robust. The higher the censoring rate the lower is the power. Source data are provided as a Source Data file.

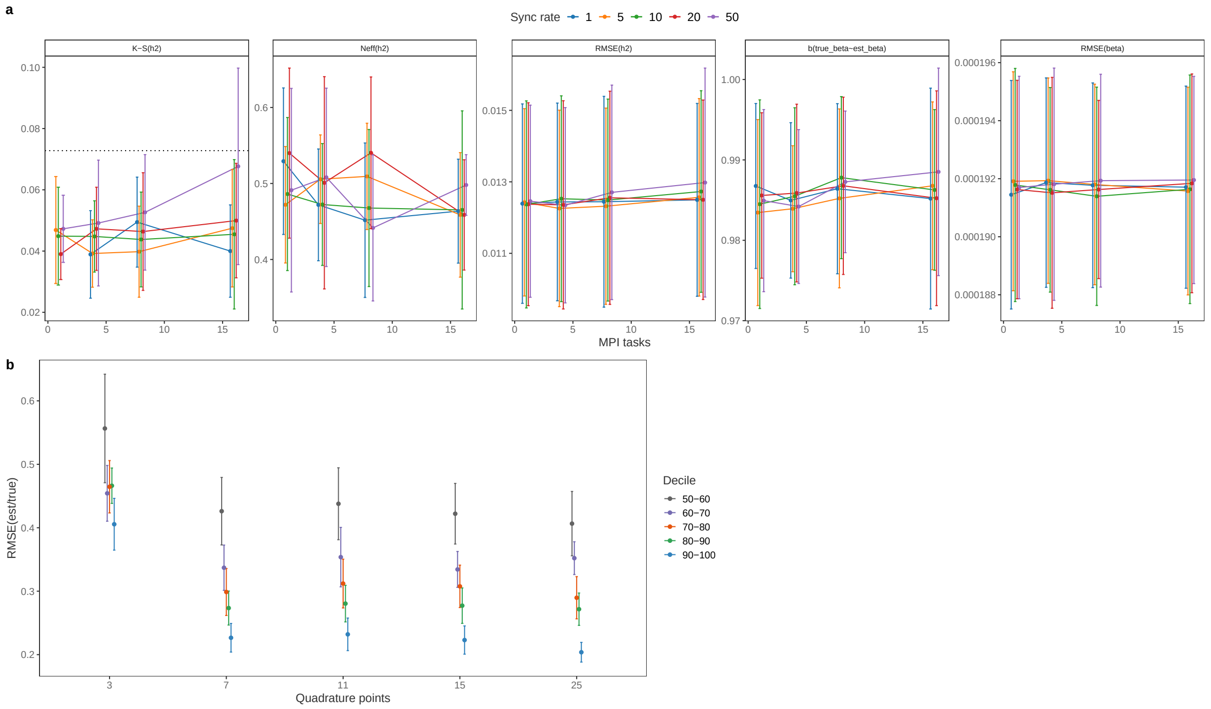

Figure S3. Computational recommendations for using BayesW. The plot centres indicate the mean and error bars indicate the standard deviation of the statistics. (a) Statistics to compare the MPI stability in various scenarios. Compared statistics: 1) Kolmogorov-Smirnov (K-S) test statistic for h^2 where every MPI setting was compared to the sequential version (one task, sync rate = 1), the dotted line indicates the critical value for the K-S statistic at the significance level of 0.01, K-S test statistic is for one-sided hypothesis; 2) Number of effective samples per one iteration for h^2 after thinning of 5; 3) RMSE for h^2; 4) Linear regression slope when regressing the true marker values on the estimated ones; 5) RMSE for β parameters. We took the chromosome 1 of the UK biobank data set that was pruned for LD of 0.9 ($M = 230,227$), with randomly sampled $N = 25,000$ individuals to make it similar to the real data setting. Phenotypes were simulated from Weibull distribution with heritability $h^2 = 0.5$ and the number of causal loci was $p = 2,500$. The models were run with three variance components (0.0001,0.001,0.01) with 10 simulations and 5 chains per MPI setting. The settings with 8 MPI tasks ($\sim 30,000$ markers per task) corresponds roughly to the setting in which full data sets are analysed. In the case where we are using very high synchronisation rate and split markers between many tasks, the estimates might deviate away from the sequential sampling results but for most settings updating markers synchronously will yield similar results compared to sequential sampling. (b) Impact of the number of quadrature points on the estimation of effect sizes. Non-zero effect sizes were grouped to deciles and then RMSE(estimated/true) was calculated within each decile. We see that using more quadrature points makes the estimation better but the improvement plateaus after some point. Source data are provided as a Source Data file.

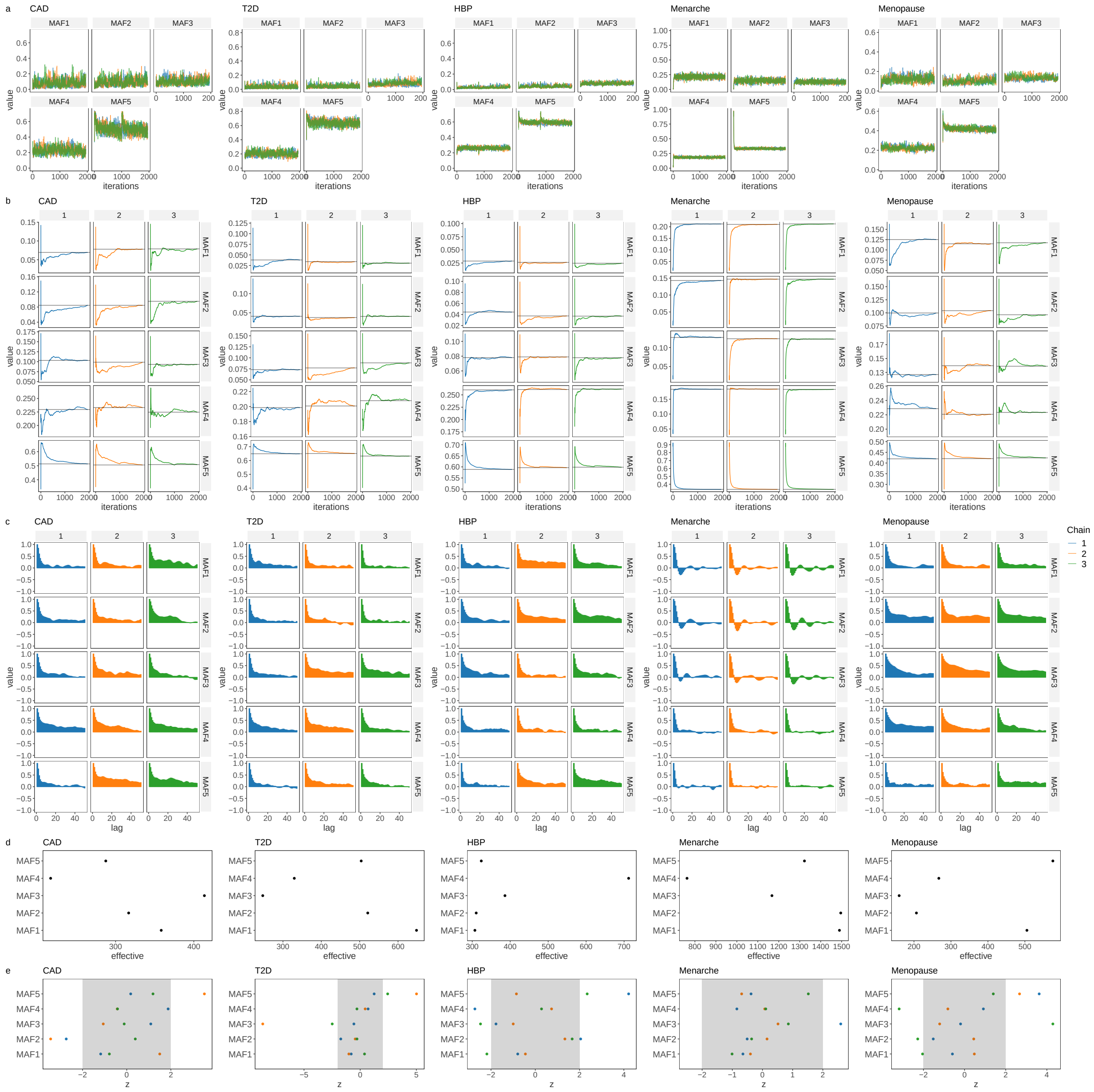

Figure S4. Convergence diagnostics of model chains for UK Biobank analysis with markers grouped into minor allele frequency (MAF) quintiles and then further subset into linkage disequilibrium (LD) quartiles. (a) Trace plot of the proportion of variance attributable to SNP markers across MAF quintiles for each trait, with colours representing the different chains; (b) a time series of the running mean of each chain, of the proportion of variance attributable to SNP markers for each MAF quintile and each trait, showing all chains approach the same mean value for each parameter; (c) lagged autocorrelation plot of each chain, for each MAF quintile and each trait; (d) effective number of uncorrelated sampled obtained for each MAF quintile and each trait. As phenotypic variance is being partitioned it is not expected that posterior estimates obtained are entirely uncorrelated; (e) Geweke z-score statistic comparing the initial part of the chain to the final part, for each MAF quintile and each trait.

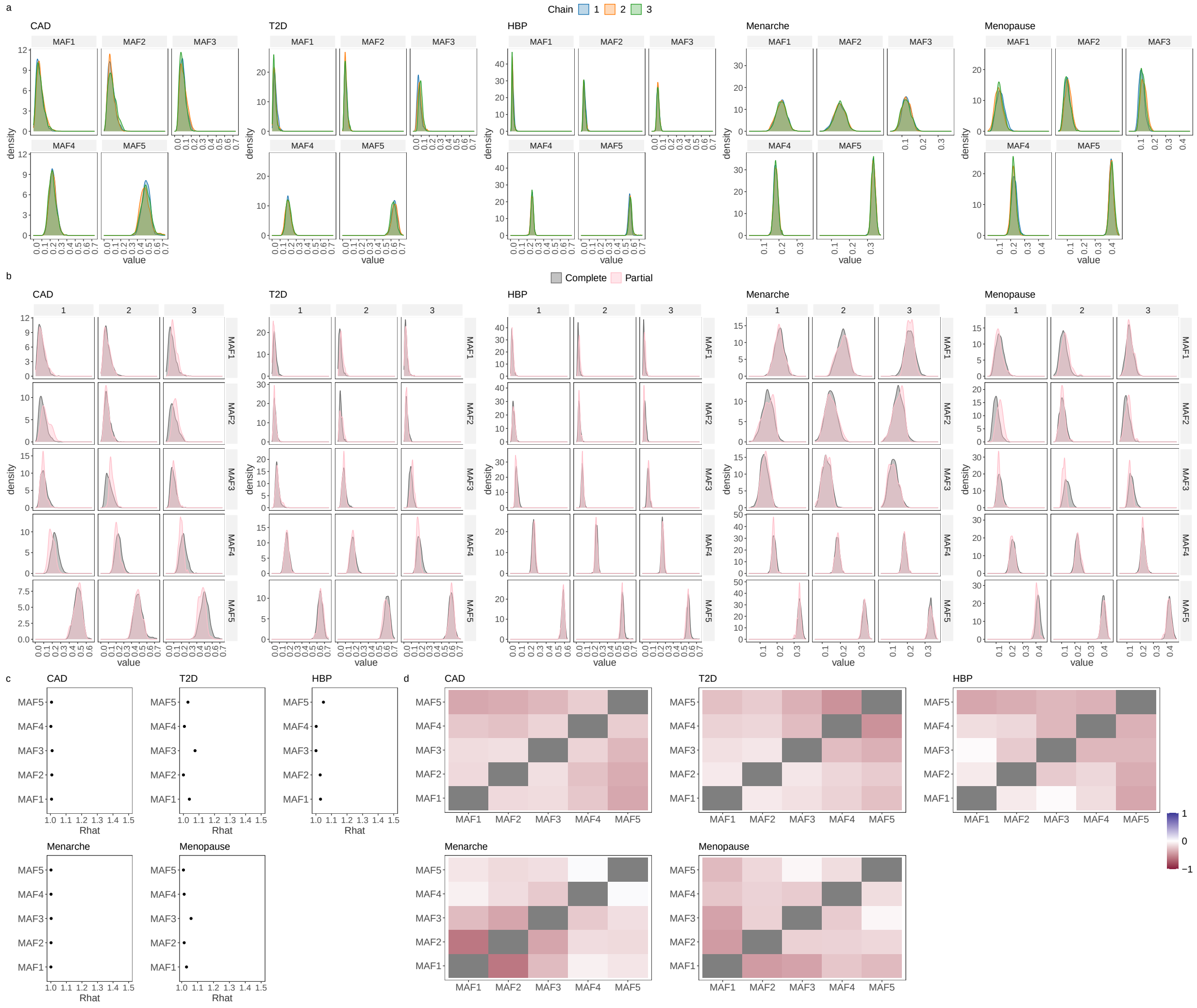

Figure S5. Convergence diagnostics of model chains for UK Biobank analysis with markers grouped into minor allele frequency (MAF) quintiles and then further subset into linkage disequilibrium (LD) quartiles.(a) Overlapped density plots to compare the target distribution by chain showing each chain has converged in a similar space, for each MAF quintile and each trait; (b) overlapped density plots comparing the last 10 percent of the chain (green), with the whole chain (pink), showing that the initial and final parts of the chain are sampling the same target distribution for each MAF quintile and each trait; (c) the potential scale reduction factor comparing the among- and within-chain variance for each MAF quintile and each trait; (d) the cross-correlation between all parameters for each MAF quintile and each trait.

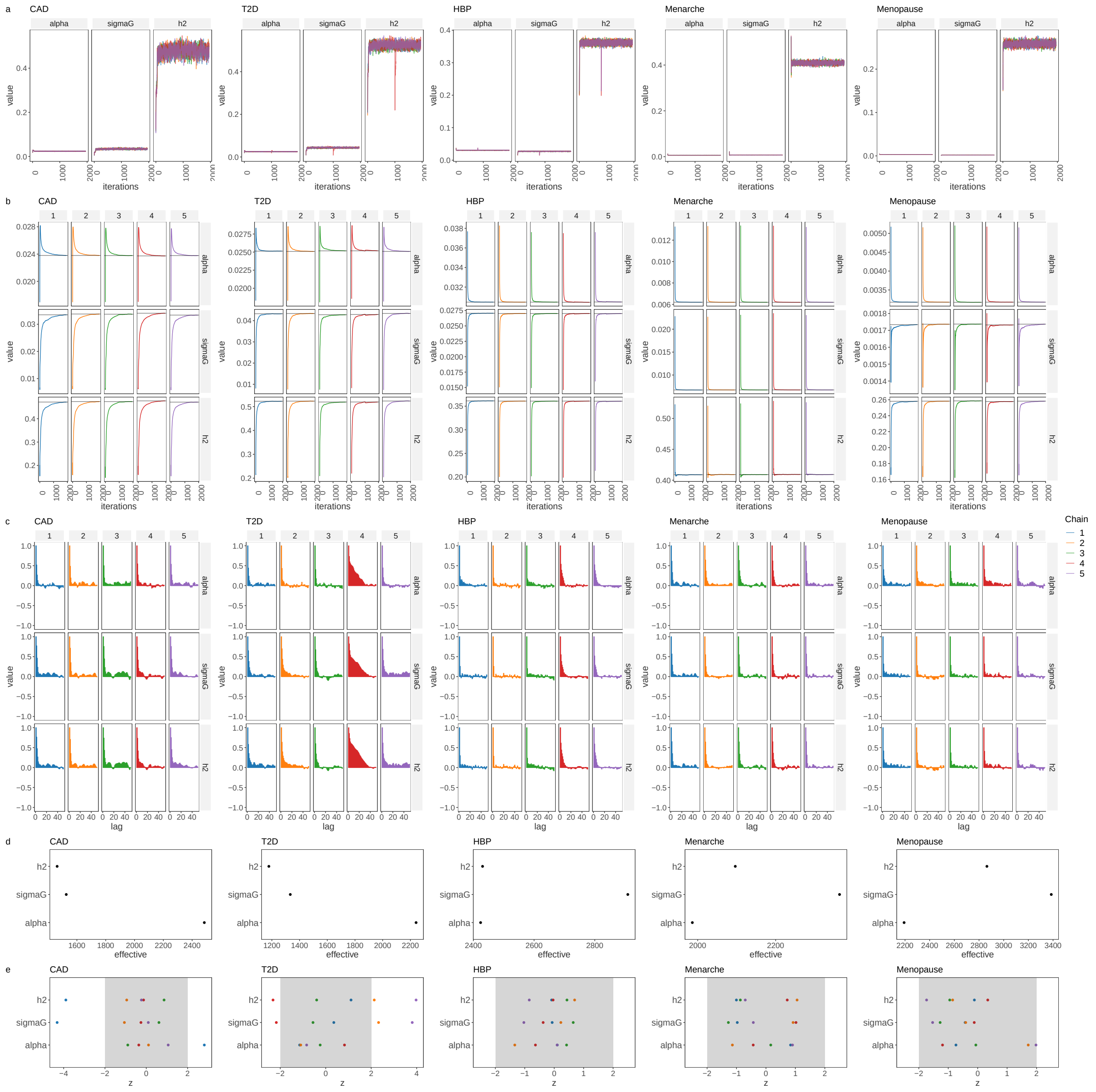

Figure S6. Convergence diagnostics of model chains for UK Biobank analysis with single marker group.(a) Trace plot of the residual variance calculated as $\pi^2/(6\alpha^2)$ labelled α, phenotypic variance attributable to SNP markers (σ^2_G), and the SNP-heritability (h^2) of each trait, with colours representing the different chains; (b) a time series of the running mean of each chain, for each trait showing all chains approach the same mean value for each parameter; (c) lagged autocorrelation plot of each chain, for each trait; (d) effective number of uncorrelated sampled obtained for each trait. As phenotypic variance is being partitioned it is not expected that posterior estimates obtained are entirely uncorrelated; (e) Geweke z-score statistic comparing the intial part of the chain to the final part, for each trait.

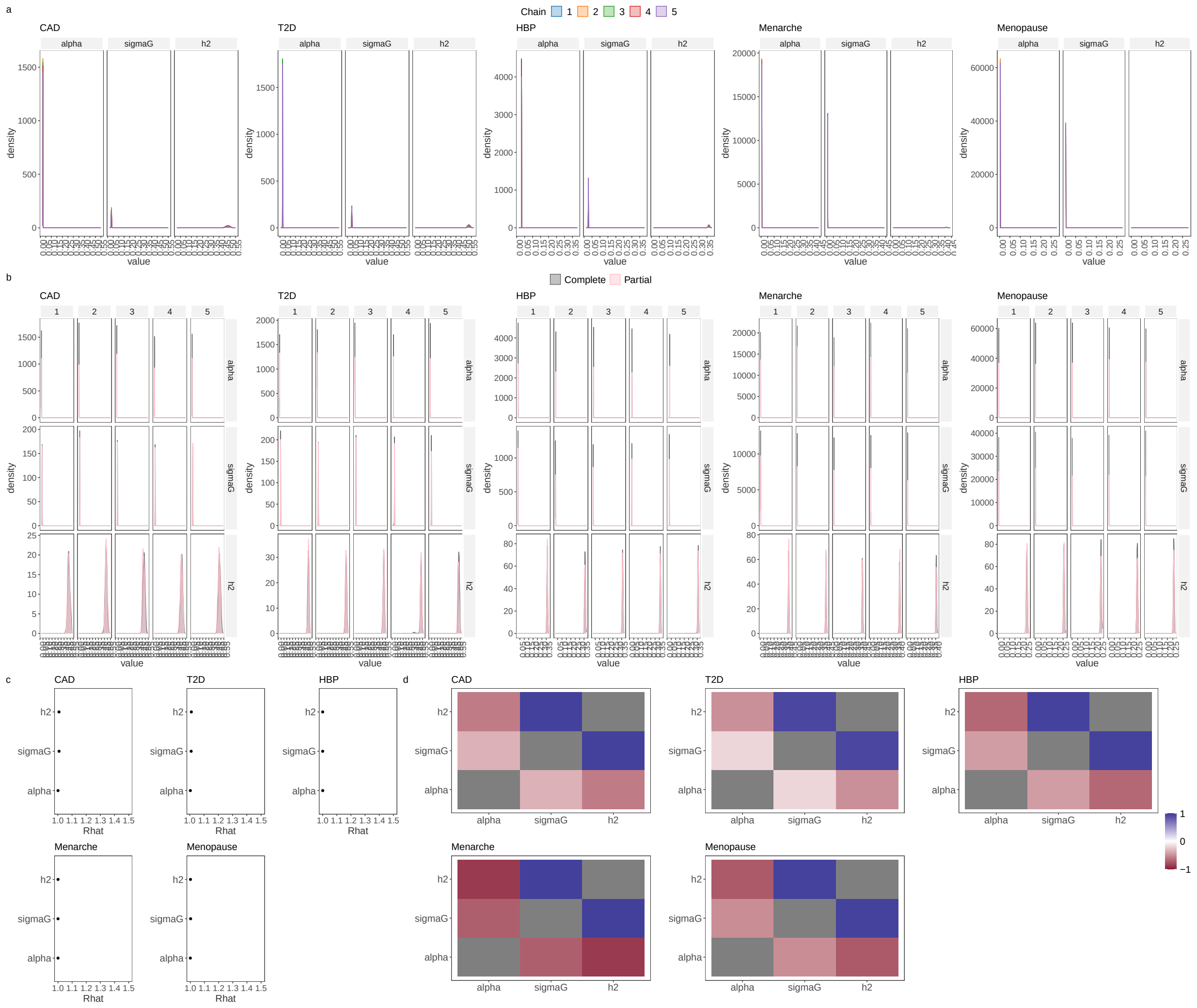

Figure S7. Convergence diagnostics of model chains for UK Biobank analysis with single marker group. (a) Overlapped density plots to compare the target distribution by chain showing each chain has converged in a similar space, for each trait, with the residual variance calculated as $\pi^2/(6\alpha^2)$ labelled α, phenotypic variance attributable to SNP markers labelled σ^2_G), and the SNP-heritability labelled h^2; (b) overlapped density plots comparing the last 10 percent of the chain (green), with the whole chain (pink), showing that the initial and final parts of the chain are sampling the same target distribution for each trait; (c) the potential scale reduction factor comparing the among- and within-chain variance for each trait; (d) the cross-correlation between all parameters for each trait.

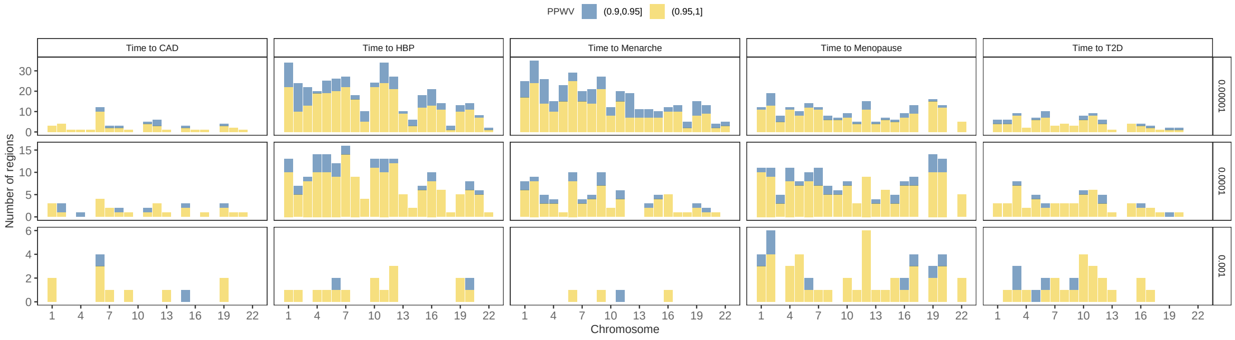

Figure S8. LD clumped region contributions to the time-to-diagnosis of CAD, HBP, T2D and age-at-menarche and age-at-menopause using no groups model. Count of LD clumped regions with high PPWV (Posterior Probability Window Variance). We conducted LD clumping procedure to partition genome into regions that have low LD between each other ($r^2 < 0.1$ between index SNPs) and then we calculated the probability that a region is explaining at least either 0.001%, 0.01% or 0.1% of the genetic variance (PPWV) using no groups model. Source data are provided as a Source Data file.

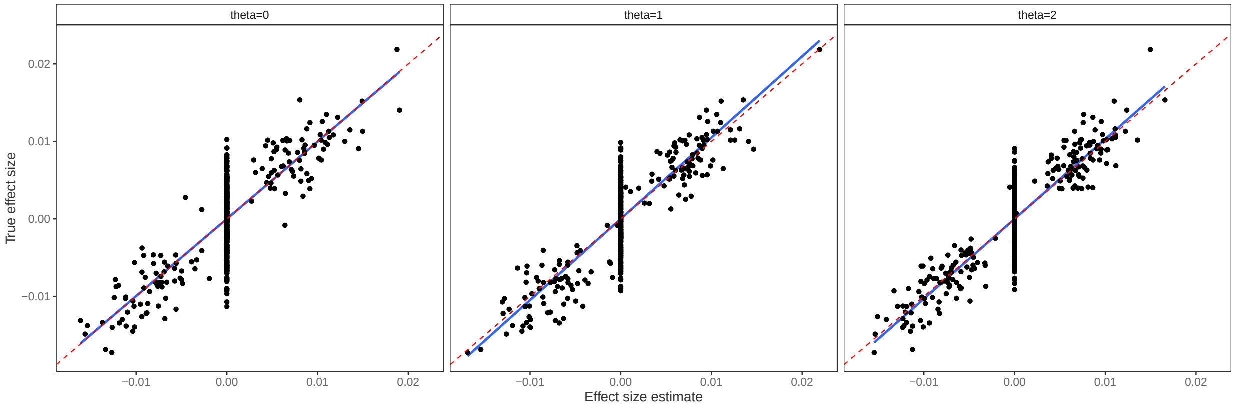

Figure S9. True effect size regressed on the BayesW estimated effect size at one iteration. Same simulation scenario as described for Figure 1c; the blue line is the estimated regression slope between true and estimated effect sizes and the red line is the slope true=estimated. Estimated slopes in the figure are 0.996, 1.048 and 1.031 for $\theta = 0, 1$ and 2 respectively. As shown in the Figure 1c we observe a slope between true and estimated effect sizes that indicates on average a very slight underestimation of the effect sizes even if the model is correctly specified ($\theta = 1$). This is likely happening due to the selected normal prior for the effect sizes yields a model that is giving ridge regression estimates that are known to slightly shrink the effect size estimates. On the other hand, if $\theta = 0$ we seemingly get a better fit for the effect sizes for the misspecified model due to the inflated hyperparameter estimate that is reducing shrinkage of the effect sizes. Source data are provided as a Source Data file.

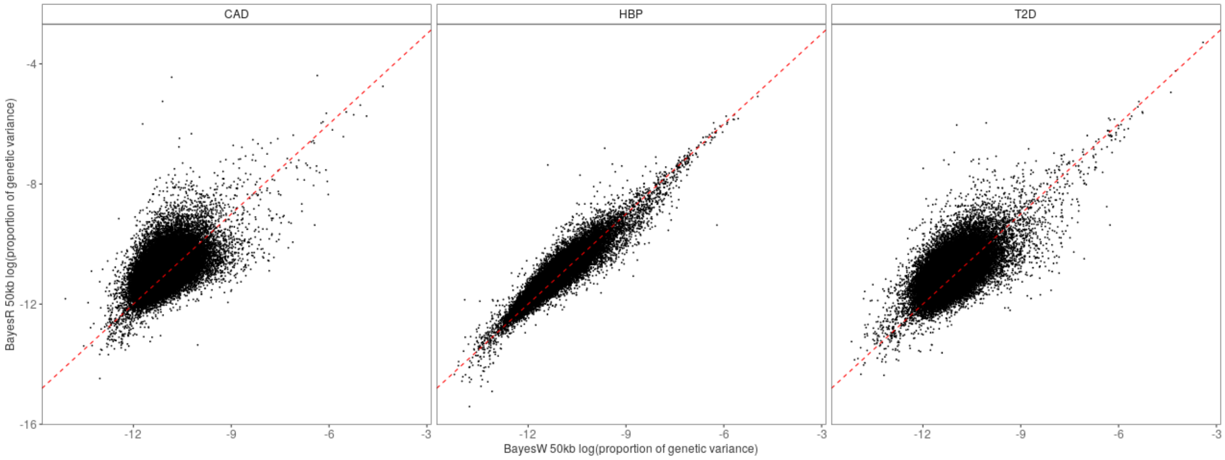

Figure S10. Logarithm of mean proportion of genetic variance explained by each 50kb region using either time-to-event model (BayesW groups) or case-control approach (BayesRR). Three analysed phenotypes were (time-to-)HBP, (time-to-)T2D and (time-to-)CAD and the analysis was conducted on unrelated UK Biobank individuals. The BayesW groups model is using 20 MAF-LD groups and BayesR model is using 36 groups based on genomic annotations and MAF-LD binning. Both modelling frameworks reach similar conclusions in terms of discovered regions: for HBP, T2D and CAD the correlation between the logarithm of results are 0.941, 0.647 and 0.554 respectively. Source data are provided as a Source Data file.

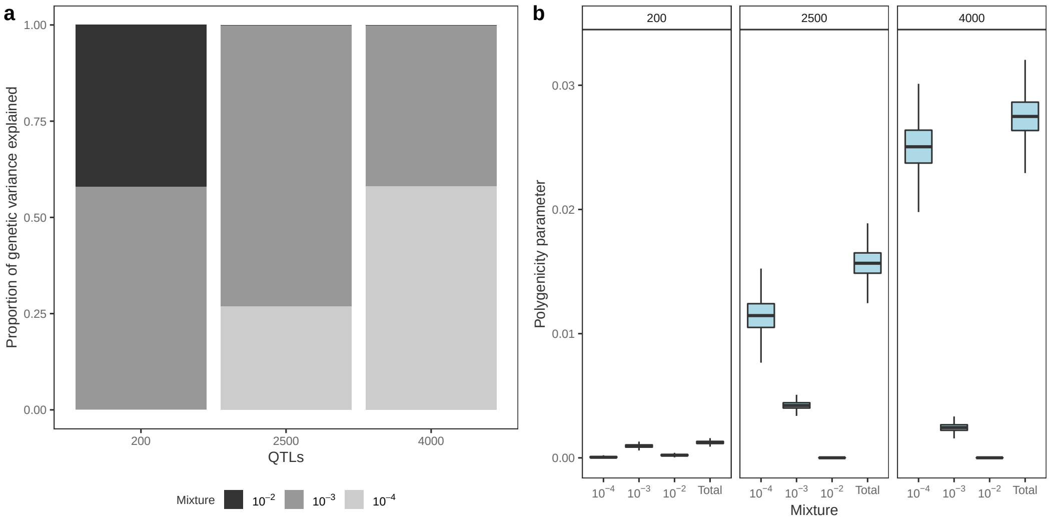

Figure S11. Variance partitioning and polygenicity for different number of causal loci. (a) The mean proportion of genetic variance explained by different mixture components given the number of causal loci. With a smaller number of causal markers and fixed heritability, we attribute more of the genetic variance to the larger mixtures 10^{-2} and 10^{-3} whereas with a higher number of causal markers and fixed heritability the genetic variance is assigned to smaller mixtures of 10^{-4} and 10^{-3}; (b) polygenicity parameter (proportion of markers with non-zero effect size) given the number causal loci. The model identifies correctly the magnitude of causal loci, a small number of causal loci results in a small number of non-zero effect size estimates and a larger number of causal loci results in a larger number of non-zero effect size estimates. Simulation setting: chromosome 1 markers (M =230,227) were used to create 10 data sets (for 10 simulations) with a different number of effect sizes (200, 2500 and 4000), in total 30 phenotypic data sets; heritability $h^2 = 0.5$, no censoring, phenotypes simulated from Weibull distribution; data from randomly selected $N = 25,000$ UK Biobank individuals. Effects were assigned to index SNPs from randomly chosen LD clumps acquired from an LD clumping procedure using $r^2 = 0.1$. In panel (b), bounds of the box show the interquartile range, centre shows the median and minimum and maximum indicate the 95% credibility interval. Source data are provided as a Source Data file.

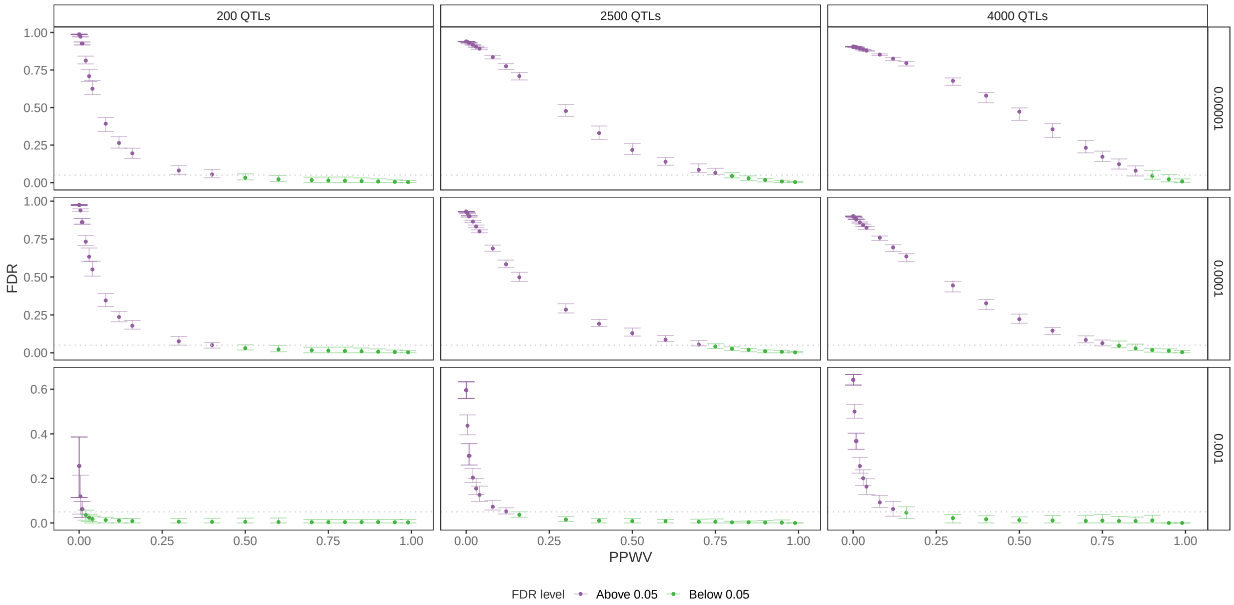

Figure S12. Relationship between PPWV thresholds and FDR throughout a different number of causal loci. LD clumps ($r^2 = 0.1$) were used as blocks for calculating PPWV (the probability that the block variance is exceeding 0.001, 0.0001 or 0.00001 of the total genetic variance). For each such clump, it was determined whether it was as a true positive or a false positive and using those the false discovery rate was calculated. Simulation setting: chromosome 1 markers (M =230,227) were used to create 10 data sets (for 10 simulations) with a different number of effect sizes (200, 2500, and 4000), in total 30 phenotypic data sets; heritability $h^2 = 0.5$, no censoring, phenotypes simulated from Weibull distribution; data from randomly selected $N = 25,000$ UK Biobank individuals. Effects were assigned to index SNPs from randomly chosen LD clumps acquired from an LD clumping procedure using $r^2 = 0.1$. The plot centres indicate the mean and error bars indicate the 95% credibility intervals of FDR. Source data are provided as a Source Data file.

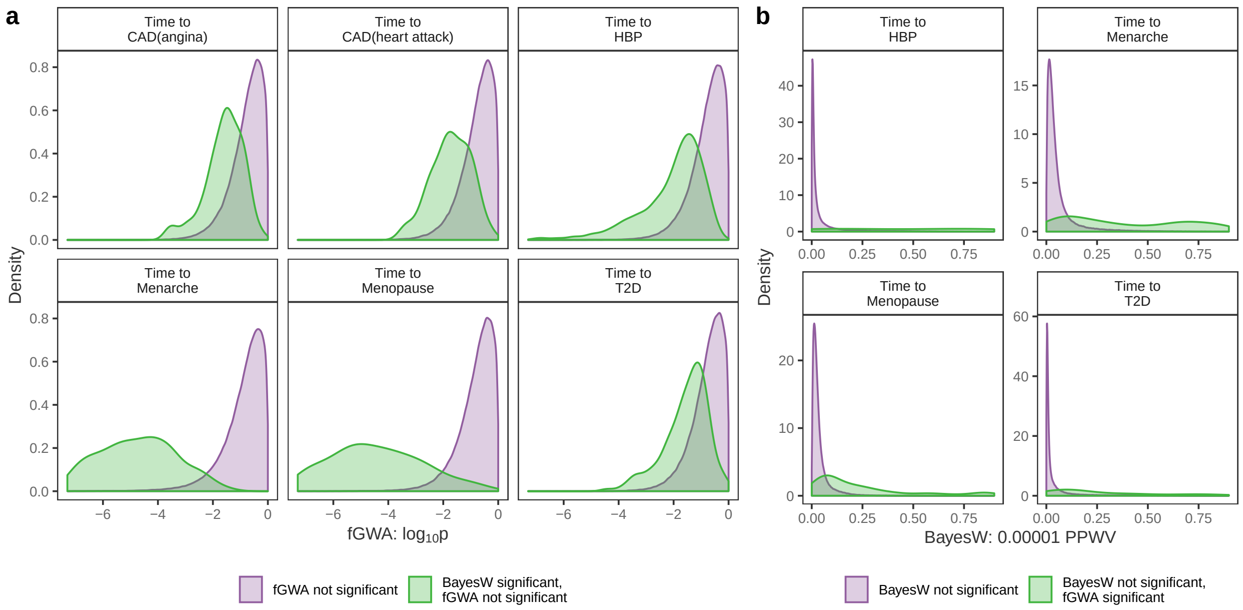

Figure S13. Densities of the discoveries missed by fastGWA or BayesW. (a) Estimated log p-value densities for all the fastGWA-non-significant LD clumps and for the fastGWA-non-significant LD clumps that were deemed significant by BayesW (a clump called significant if PPWV of a clump ≥ 0.9, PPWV-based test is one-sided as it is using squares of effect size estimates). LD clumps that were called significant by BayesW tend to be shifted left in the figure indicating that fastGWA might have missed those effects due to insufficient power. (b) Estimated PPWV densities for all the BayesW-non-significant LD clumps and for the BayesW-non-significant clumps that were deemed significant by fastGWA. p-values from fastGWA are calculated from $\chi^2_{df=1}$ statistics for the null hypothesis that a marker does not have an effect and the marker or clump was called significant if the corresponding p-value was lower than the multiple testing adjusted significance threshold of $5 \cdot 10^{-8}$. The minimum p-value in a clump was used to determine the significance of a clump.

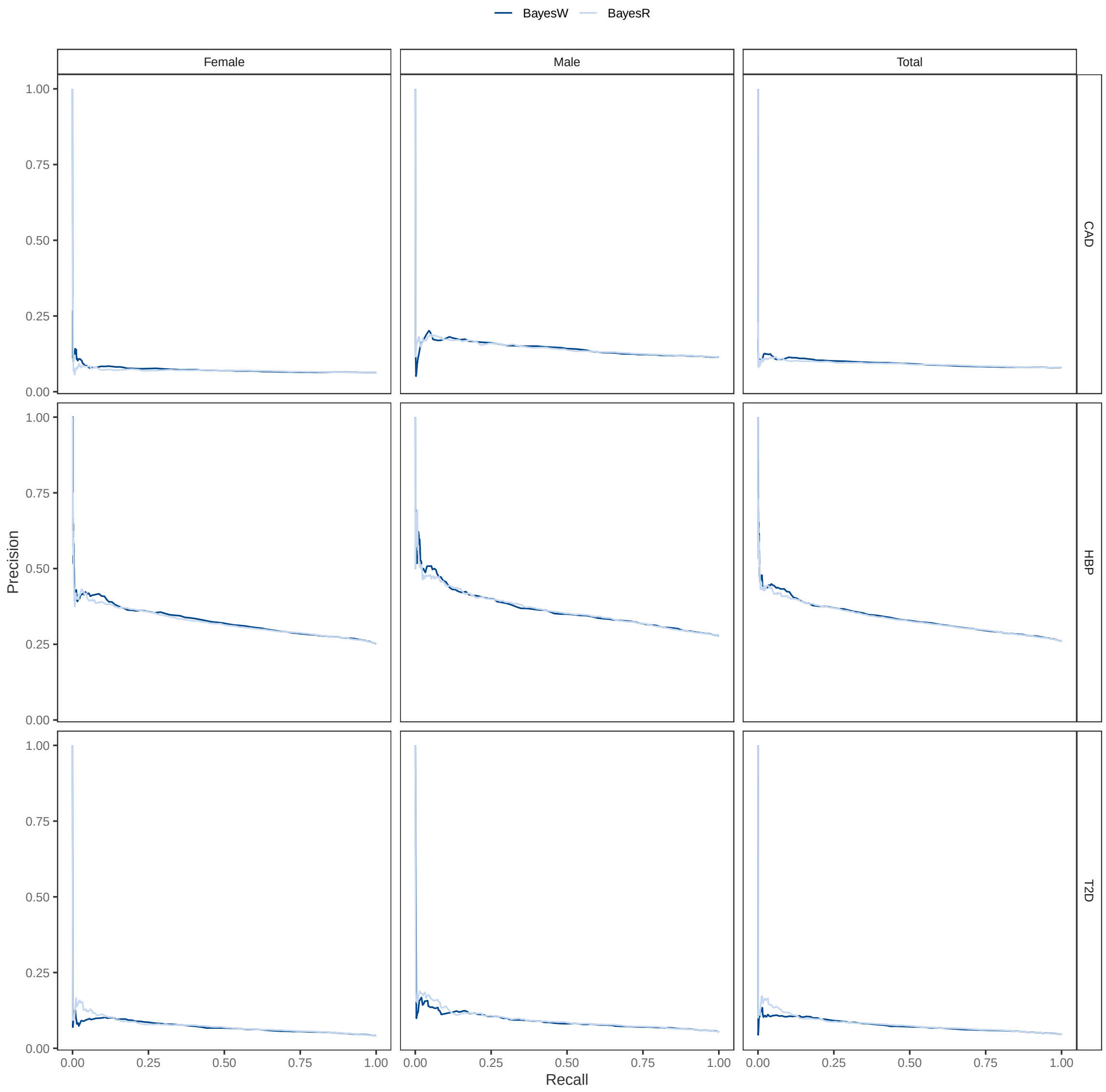

Figure S14. Precision-recall curves for predicting binary phenotypes (CAD, HBP, T2D) in Estonian biobank using either BayesR or BayesW model. Both models were trained on UK biobank data but BayesR model was using binary case-control phenotypes treating them as continuous variables and BayesW model was using respective time-to-diagnosis phenotypes. Precision-recall curves were drawn separately for females, males and all of the people (Total). Source data are provided as a Source Data file.

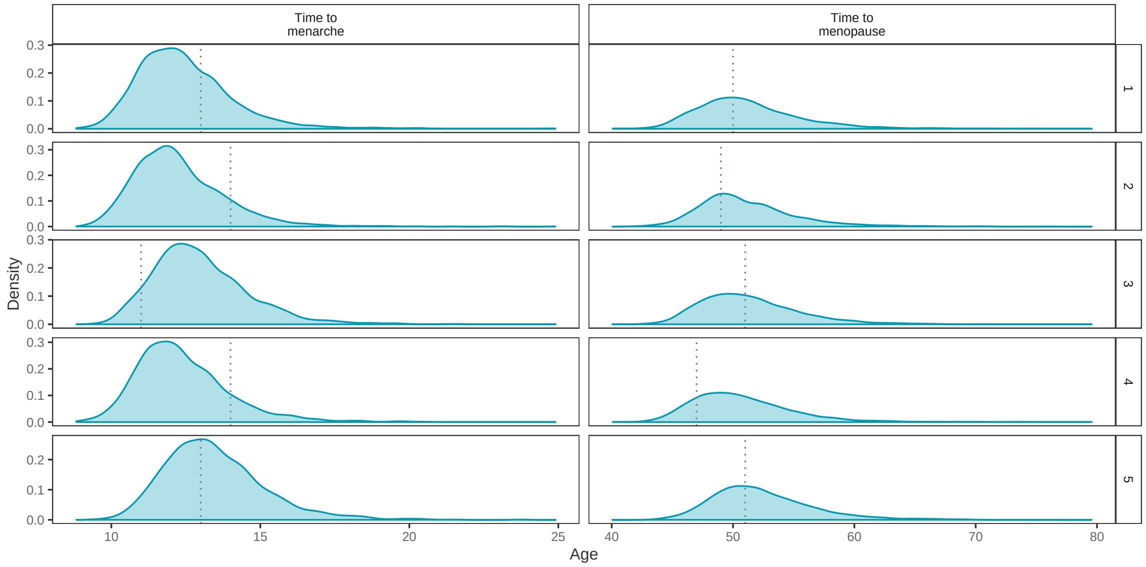

Figure S15. Posterior predictive distributions from BayesW for five random individuals from the Estonian biobank for time-to-menarche and time-to-menopause. BayesW enables calculating individual posterior predictive distributions, dotted lines indicate the true age at event. For age-at-menarche and age-at-menopause 94.8% and 92.3% of the true phenotypes from the Estonian biobank lie within 95% credibility intervals of those predictive distributions.
